# Infective Juveniles of the Entomopathogenic Nematode, *Steinernema feltiae* Produce Cryoprotectants in Response to Freezing and Cold Acclimation

**DOI:** 10.1371/journal.pone.0141810

**Published:** 2015-10-28

**Authors:** Farman Ali, David A. Wharton

**Affiliations:** Department of Zoology, University of Otago, P.O. Box 56, Dunedin, New Zealand; University of California at Berkeley, UNITED STATES

## Abstract

*Steinernema feltiae* is a moderately freeze-tolerant entomopathogenic nematode which survives intracellular freezing. We have detected by gas chromatography that infective juveniles of *S*. *feltiae* produce cryoprotectants in response to cold acclimation and to freezing. Since the survival of this nematode varies with temperature, we analyzed their cryoprotectant profiles under different acclimation and freezing regimes. The principal cryoprotectants detected were trehalose and glycerol with glucose being the minor component. The amount of cryoprotectants varied with the temperature and duration of exposure. Trehalose was accumulated in higher concentrations when nematodes were acclimated at 5°C for two weeks whereas glycerol level decreased from that of the non-acclimated controls. Nematodes were seeded with a small ice crystal and held at -1°C, a regime that does not produce freezing of the nematodes but their bodies lose water to the surrounding ice (cryoprotective dehydration). This increased the levels of both trehalose and glycerol, with glycerol reaching a higher concentration than trehalose. Nematodes frozen at -3°C, a regime that produces freezing of the nematodes and results in intracellular ice formation, had elevated glycerol levels while trehalose levels did not change. *Steinernema feltiae* thus has two strategies of cryoprotectant accumulation: one is an acclimation response to low temperature when the body fluids are in a cooled or supercooled state and the infective juveniles produce trehalose before freezing. During this process a portion of the glycerol is converted to trehalose. The second strategy is a rapid response to freezing which induces the production of glycerol but trehalose levels do not change. These low molecular weight compounds are surmised to act as cryoprotectants for this species and to play an important role in its freezing tolerance.

## Introduction

Water plays a vital role in the structural and functional stability of macromolecules and maintains the integrity of lipid membranes in biological systems [1]. When water is removed due to osmotic dehydration or freezing the membrane may become permeable to solvents, membranes may fuse and membrane particles can aggregate resulting in fatal damage [2]. Some nematodes are anhydrobiotic (survive without water); such as *Aphelenchus avenae* [3], *Anguina tritici* [4], *Caenorhabditis elegans* dauers [5] and *Ditylenchus dipsaci* [6], and some are freeze tolerant; such as *Steinernema feltiae* [7] and *Panagrolaimus davidi* [8]. Most of these nematodes produce low molecular weight compounds in response to dehydration or freezing stress which act as cryoprotectants or anhydroprotectants [9,10,11].

Cryoprotectants are compounds that protect the organism from chilling and freezing injury and thereby enhances its cold tolerance [12]. These include sugars such as trehalose, glucose, fructose, and polyhydric alcohols such as glycerol, sorbitol, myo-inositol, ethylene glycol, ribitol, erythritol and inositol [13]. These cryoprotectants depress the melting point of the body fluids and thus decrease the amount of ice formed [14]. Trehalose is believed to assist the nematodes with their short-term freezing stress, while long-term freezing survival may be attributed to the presence of a recrystallization inhibition protein [11] which helps to stabilize the structure and size of the ice crystals after their formation [15]. Glycerol has also been shown to increase the freezing tolerance of nematodes [16] and to permeate the membrane once the water is lost [17]. Entomopathogenic nematodes have been reported to accumulate cryoprotectants such as trehalose and glycerol in response to low temperature [9,18,19,20,21,22]. Accumulation of these cryoprotectants in freeze tolerant nematodes are induced by either cold acclimation [20] or cold and heat shock [19] prior to their anticipated freezing. However, there is no report on the synthesis of cryoprotectants in nematodes in response to freezing, as has been reported in some freezing tolerant earthworms [23].

In the present study, we have examined *S*. *feltiae* for potential cryoprotectants following low-temperature acclimation and freezing regimes that result in cryoprotective dehydration (freezing at -1°C) or intracellular freezing (freezing at -3°C) [7]. Cryoprotective dehydration was first described in earthworm cocoons and describes a situation where ice formation in the soil or water surrounding an animal does not produce freezing within its body. Its body contents thus remain liquid and water is lost to the surrounding ice, due to the difference in vapour pressure between the nematode’s body fluids and the surrounding ice, so that the animal dehydrates [24]. The Antarctic nematode *Panagrolaimus davidi* was the first nematode shown to survive intracellular freezing [24] but other nematodes, including *S*. *feltiae*, have more limited abilities to do so [7,25].

Since the cold tolerance mechanism and the ability of *S*. *feltiae* to survive freezing varies with different acclimation and freezing manipulations [26], the main aim of this study was to compare the cryoprotectant profiles of this nematode after these treatments. In addition to cryoprotectants induced by cold acclimation, this study for the first time describes the accumulation of cryoprotectants in nematodes in response to the freezing process *per se*.

## Methods

### Nematode culture, freezing and acclimation regimes

Infective juveniles of *S*. *feltiae* were locally-collected strains obtained from AgResearch Lincoln. The species is cosmopolitan, temperate in nature and was collected from the Lincoln area in New Zealand. The culture was maintained in the last instar larvae of the bee wax moth, *Galleria mellonella* at 22°C. Third-stage infective juveniles of *S*. *feltiae* were harvested from dead *G*. *mellonella* larvae in White traps [27], and passed through two layers of tissue paper to obtain active nematodes. Nematodes were either acclimated at 5°C for two weeks or processed fresh after harvesting at room temperature as non-acclimated controls. Test samples were a 1 ml suspension of about 100 nematodes in artificial tap water [28]. A third regime was freshly harvested infective juveniles subjected to freezing at −1°C and left overnight in the refrigerated circulator. This regime results in cryoprotective dehydration [26]. A fourth regime was freshly harvested infective juveniles frozen at −3°C and held for 75 minutes, resulting in intracellular freezing of the infective juveniles [7]. The nematodes were removed from the refrigerated circulator in the frozen state after holding for 75 minutes and processed immediately.

### Sugars and polyols analysis

Low molecular weight cryoprotectants were analysed by gas chromatography, as described by Wharton *et al* [11]. The non-acclimated infective juveniles were washed in artificial tap water [28] and centrifuged to get a concentrated pellet. Artificial tap water was added to make up the suspension to just over 1 ml. The weight of a 10 μl subsample of each regime was determined after drying in an oven to calculate the total dry weight of the nematode sample. Exactly 1 ml of nematode suspension was then either frozen at −3°C (producing intracellular freezing, subjected to overnight freezing at −1°C (cryoprotective dehydration), or centrifuged (non-acclimated control at room temperature) and the supernatant removed. Then 20 μl dulcitol (as an internal standard) and 1 ml extraction mixture (cold chloroform/methanol/dH_2_O; 8:3:1 v/v) was added to each sample. The samples were then transferred to a glass homogenizer and homogenized for 15 minutes on ice until the nematodes disrupted completely. The homogenate was then transferred to a glass centrifuge tube followed by 1 ml of dH_2_O used to rinse the homogeniser. The homogenate was centrifuged at 3000 rpm for 10 minutes and the top aqueous layer taken. The aqueous portion was then passed through a Bond Elute SCX column (Varian SPP: preconditioned by passing through 2 ml methanol, then 2 ml methanol:0.1 M HCl, and then 2 ml 0.1M HCl) and collected in Eppendorf tubes. The column was rinsed with 0.5 ml dH_2_O, which was added to the sample. The sample, now containing everything minus lipids and proteins, was dried down under nitrogen in a heating block at 40°C. When the sample was small enough, it was transferred to a chromatography vial. The completely dried sample in the vial was capped, used immediately or stored in a desiccator until use.

Polyols and sugars were converted to their trimethylsilyl derivatives by adding 20 μl Silprep (Alltech, Deerfield IL). The vial was sealed with parafilm as the Silprep is sensitive to water, rotated slowly to dissolve the sample and allowed to incubate for 15 minutes at room temperature. Five microlitres of the sample was injected into an Econocap EC-5 capillary column (Alltech, New Zealand) on a gas chromatograph (GC; Agilent 6890N Series gas chromatography system; Agilent Technologies, Wilmington, USA) controlled by GC Chemstation software run on a PC. Polyols and sugars were identified and quantified according to their retention times and peak areas with reference to standards.

The concentrations of all the three cryoprotectants in each treatment were determined and compared with the controls. Total carbohydrates measured were also calculated for each treatment and compared between treatments and with the control. Either one way analysis of variance (one-way ANOVA) or multivariate analysis of variance (MANOVA) was carried out using the Statistical Package for Social Sciences (SPSS) ver. 15.0 [29] to see if the carbohydrate concentrations measured were significantly different among the treatments. Means were separated using Tukey’s multiple comparison test.

## Results

**Fig 1:**
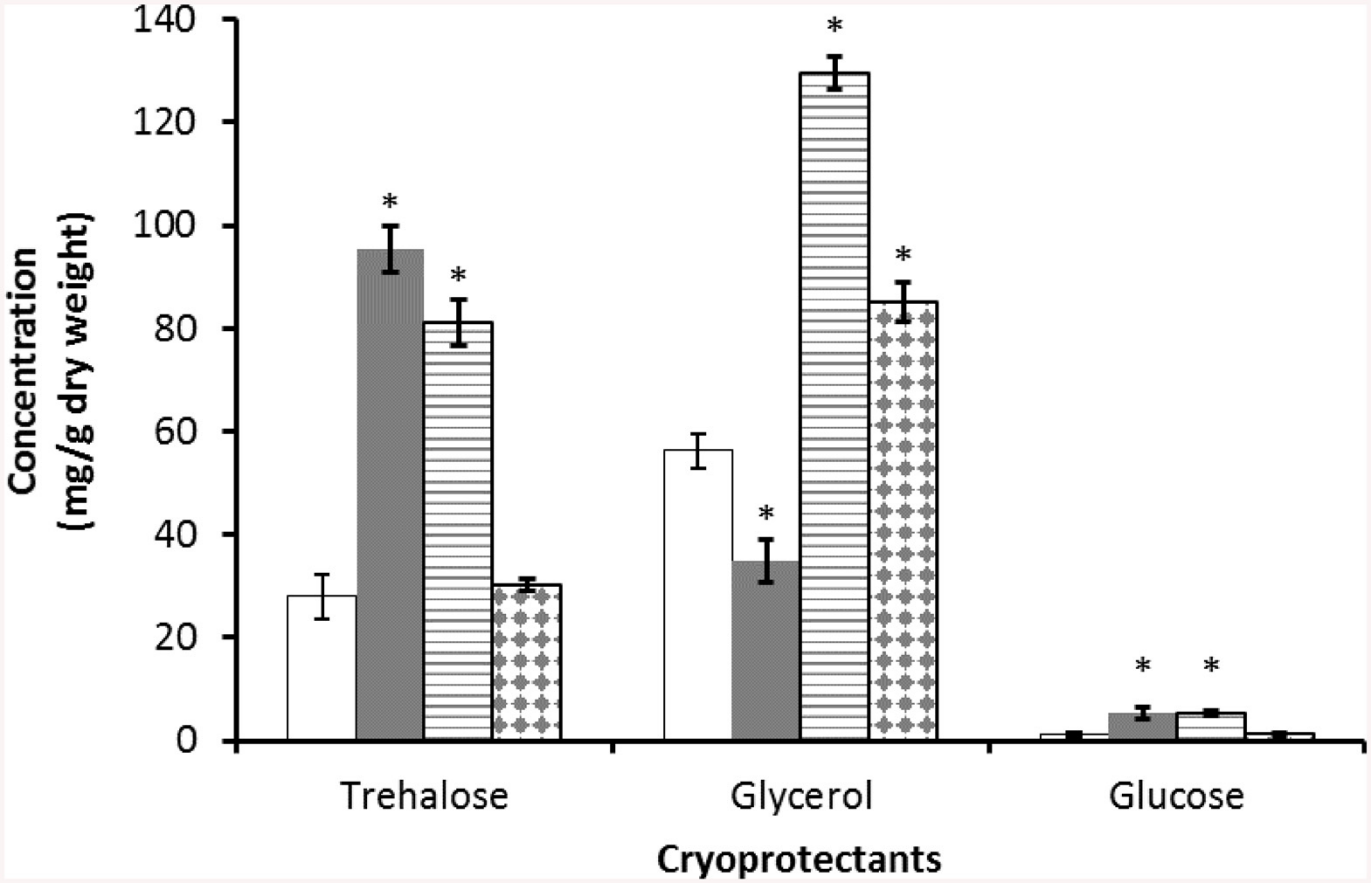
Concentration of sugars and polyol accumulated in the infective juveniles of *S*. *feltiae* in non-acclimated controls (open bars), after two weeks’ acclimation at 5°C (closed bars), after freezing overnight at −1°C (cryoprotective dehydration: horizontal line bars) and after freezing at −3°C for 75 minutes (intracellular freezing: diamond bars). An asterisk above the bars of acclimated and frozen treatments indicates a significant difference from the non-acclimated control (P<0.05). Error bars represent standard errors of 3 replicates.

Three carbohydrates trehalose, glycerol and glucose were detected by gas chromatography in the infective juveniles of *S*. *feltiae*. Trehalose and glycerol were the major cryoprotectants detected, with glucose being a minor component. The initial amount of glycerol (56.32±3.8 mg g^−1^ dry weight, 193 mM) was significantly higher than that of trehalose (27.94±3.0 mg g^−1^ dry weight, 23 mM) and glucose (1.1±0.2 mg g^−1^ dry weight, 2 mM) in the non-acclimated controls (P<0.05). Nematode samples acclimated for two weeks at 5°C have significantly increased levels of trehalose and glucose, compared with non-acclimated controls (P<0.05). However, glycerol levels dropped significantly (P>0.05) (Fig 1).

In nematode samples frozen overnight at −1°C (cryoprotective dehydration), there was a significant increase in the concentration of all three carbohydrates (P<0.05) in comparison with non-acclimated controls. Trehalose increased three-fold, compared to a two-fold increase in glycerol. However, the total concentration of glycerol was higher than that of trehalose (Fig 1). Freezing at -3°C (intracellular freezing) induced the accumulation of glycerol but not of trehalose or glucose. In nematodes undergoing intracellular freezing, the concentration (81±4.1 mg g^−1^dry weight, 68 mM) of glycerol was significantly higher (P<0.05) than that in non-acclimated controls, but trehalose and glucose concentrations did not change significantly (P>0.05) (Fig 1).

Glycerol concentrations differed significantly (P<0.05) in all three treatments, with the maximum concentration (Fig 1, 129.6±5.8 mg g^−1^ dry weight, 444 mM) in samples undergoing cryoprotective dehydration, followed by the concentration in nematodes undergoing intracellular freezing.

The level of trehalose reached its maximum value (95.4±4.4 mg g^−1^ dry weight, 80 mM) in nematodes acclimated for two weeks at 5°C (Fig 1), this was not significantly different to that of samples undergoing cryoprotective dehydration but it was significantly higher than that of samples undergoing intracellular freezing. Glucose levels were very low in nematodes undergoing intracellular freezing and reached their highest levels (5.4±0.5 mg g^−1^ dry weight, 9 mM) in samples undergoing cryoprotective dehydration.

**Fig 2:**
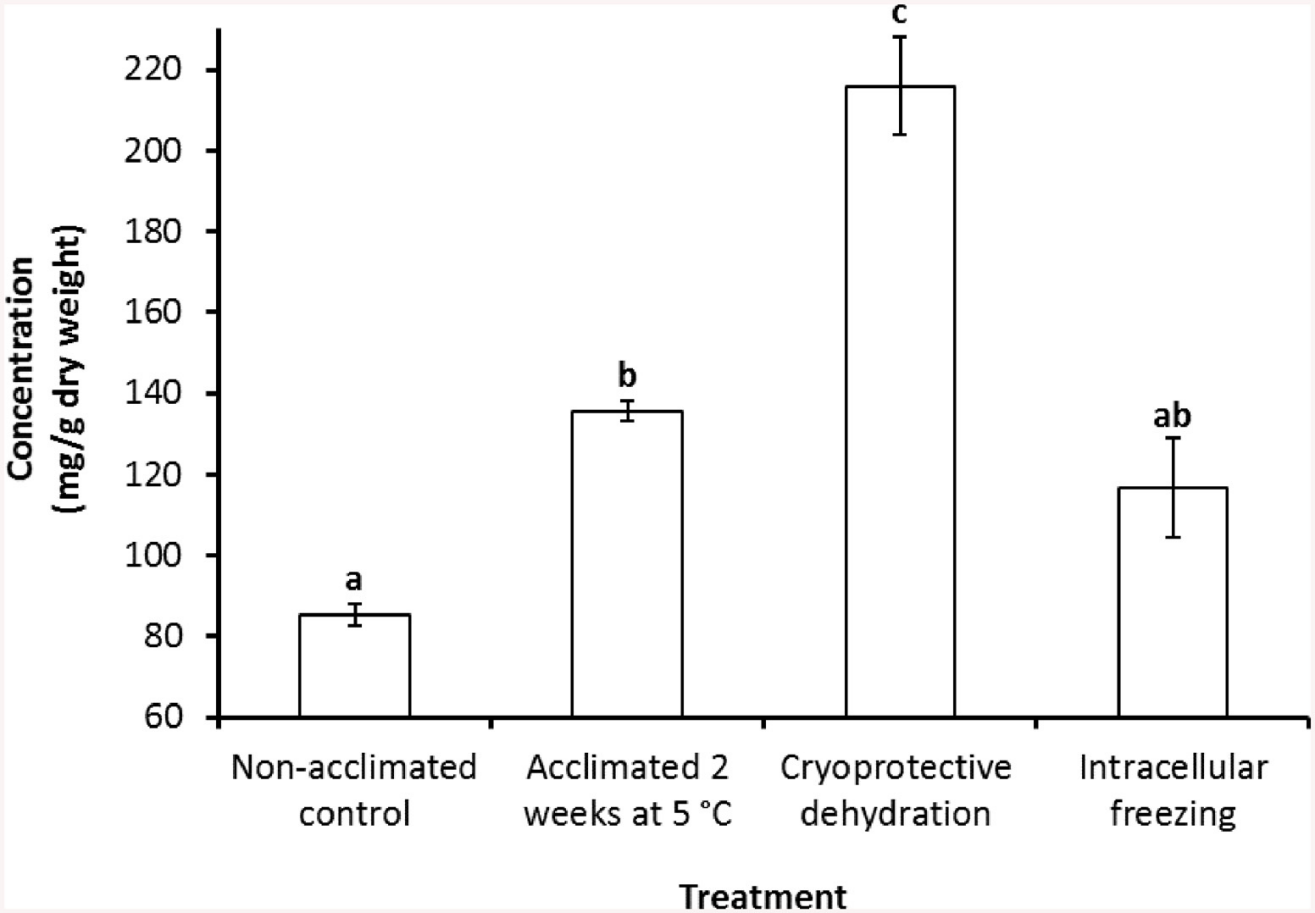
Concentration of total carbohydrates measured in the infective juveniles of *S*. *feltiae* after exposing them to various acclimation and freezing regimes. Different small letters above the bars indicate that treatments are significantly different (P<0.05). Error bars represent standard errors of 3 replicates.

The concentrations of total carbohydrates measured in all the treatments, with the exception of samples undergoing intracellular freezing, was significantly higher (P<0.05) than that in the non-acclimated controls (Fig 2). The maximum total amount of carbohydrates measured (215.9±12.2 mg g^−1^ dry weight) was recorded in nematode samples undergoing cryoprotective dehydration which was significantly different to those in the other treatments (P<0.05). There was no significant difference in the level of total carbohydrates measured between samples acclimated for 2 weeks at 5°C, and samples undergoing intracellular freezing (P>0.05).

## Discussion

Two sugars (trehalose, glucose) and a polyol (glycerol) were detected in the infective juveniles of *S*. *feltiae*. Trehalose and glycerol were in high concentrations and function as cryoprotectants in many organisms [13]. Both overnight freezing at −1°C (cryoprotective dehydration) and freezing at -3°C for 75 min (intracellular freezing) induced the accumulation of glycerol, but long-term acclimation (two weeks at 5°C) significantly decreased glycerol levels. Trehalose, in contrast increased to 95.4±4.4 mg g^−1^, 80 mM after two weeks at 5°C. This concentration is close to that previously reported from *S*. *feltiae* (82.28±4.4 mg g^−1^dry weight) and other entomopathogenic nematodes acclimated at 5°C for a week [9,20]. The decline in the glycerol level after two weeks acclimation at 5°C suggests that glycerol might be converted to glycogen and then to trehalose via the pathway from glycogen to trehalose [13], which is suggested by the elevated level of trehalose after two weeks’ acclimation at 5°C.

Glycerol concentrations increased on intracellular freezing but trehalose concentrations did not change. This is the first study on nematodes that has demonstrated cryoprotectant synthesis in response to freezing (in addition to acclimation). In the moor frog *Rana arvalis* [30] and the wood frog *Rana sylvatica* [31] it has been noted that ice nucleation triggers the synthesis of glucose during freezing. The concentration of glucose was higher in frozen frogs than it was in the unfrozen supercooled state. Some freezing tolerant earthworms also synthesize glucose in response to freezing [26]. In a freeze tolerant slug glucose accumulation is also triggered by freezing [32]. In the brown tree frog *Litoria ewingii*, the level of glycerol and not glucose increases upon freezing [33], as seen in the present study on nematodes. However, in freeze tolerant reptiles cryoprotectants are poorly developed [34]

The significant increase of glycerol but not trehalose during freezing suggests that glycerol plays a more important role than trehalose in the survival of nematodes once they are frozen. The glycerol response is rapid in response to dehydration and it quickly equilibrates the osmotic pressure [17]. Glycerol is a penetrating cryoprotectant [12] and readily permeates across membranes [35]. Therefore, its role during freezing could be more important than trehalose, which is a non-penetrating cryoprotectant [12]. Trehalose does not change during freezing and thus its role as a stress protectant is more likely to be before or on the onset of freezing. However, the presence of trehalose in the frozen nematodes suggests that its cryoprotective properties may still operate in the freezing process *per se*; nevertheless the concentration is less than that of glycerol. Trehalose is an important cryoprotectant but does not increase during the freezing process, suggesting that some other factors could be involved in coping with long-term freezing stress in this nematode after ice formation. *S*. *feltiae* is a freeze tolerant species and can survive intracellular freezing [7]. For a freeze tolerant species recrystallization or the migration of still-liquid salty domains could be quite damaging [36,37]. However, this species has been shown to have small ice crystals post freezing, and may thus, exhibit recrystallization inhibition activity which is likely to be responsible for the long term survival of freezing [7].

The difference in cryoprotectant concentrations after different acclimation or freezing regimes could also be due to the different temperatures (−1, −3, +5°C) used in the treatments tested in the present study. However, trehalose accumulation was more time than temperature dependent as there was no significant difference in trehalose concentration between nematodes undergoing cryoprotective dehydration or acclimated at 5°C for two weeks. Also in our previous experiments there was a significant improvement in survival when the exposure time of nematodes at −1°C was extended from 75 minutes to overnight [26]. During this extended exposure to the same temperature, the nematodes may accumulate more cryoprotectants, as an acclimation response to low temperature, which accounts for the difference in their survival.

Since the nematodes themselves do not freeze at −1°C [7], this extended exposure of nematodes may also result in loss of water from the nematode body due to cryoprotective dehydration [26,38]. Thus the accumulation of cryoprotectants at −1°C could be a response to both desiccation and low temperature. Dehydration results in the synthesis of desiccation protectants such as trehalose and glycerol from glycogen [39] or from lipids [35]. Freezing survival is correlated with the elevated levels of trehalose and glycerol accumulated when the nematodes are acclimated slowly over an extended period of time (~ 14 hours) before freezing. In a recent study, Shapiro et al [40] exposed various entomopathogenic nematodes including *S*. *feltiae* to freezing and desiccation stress but could not find any correlation between desiccation and freezing tolerance. However, their freezing regime and rate of cooling was different than those used in the present study.

The synthesis of trehalose continues, as long as the nematodes are unfrozen, with glycerol being converted into trehalose via glycogen. Trehalose and glycerol have been widely reported as cryoprotectants in animals [13] including several nematode species [41,42,43]. These cryoprotectants act colligatively by replacing the water and thus decreasing the amount of ice formed in the body [44]. They depress the melting point and thus lower the supercooling point, enabling the nematode to avoid freezing in some species. This could prevent the infective juveniles of *S*. *feltiae* from freezing at high sub-zero temperatures, such as −1°C [26]. Cryoprotectants can also act non-colligatively by protecting cell membranes and proteins from denaturation, maintaining the osmotic balance [45]. Glycerol as a cryoprotectant was first reported by Salt [46] from insects. Grewal and Jagdale [20] correlated cold acclimation-induced trehalose accumulation with survival in three species of entomopathogenic nematodes, including *S*. *feltiae* both at 5 and 25°C. The amount of trehalose was high at 5°C [9]. *S*. *kushidai* also accumulates 1.4% dry weight trehalose when acclimated at 5°C for 20 days [9]. The freeze tolerant Antarctic nematode *Panagrolaimus davidi* also elevates trehalose but not glycerol levels when acclimated at 5°C [11]. Qiu and Bedding [21] showed that *S*. *carpocapsae* and four other species of entomopathogenic nematodes accumulated more trehalose at 5°C than at any other temperature, thus trehalose accumulation in response to cold stress could be a common characteristic of the infective juveniles of entomopathogenic nematodes. The present study supports this notion.

*Steinernema feltiae* thus, has two strategies of cryoprotectant accumulation: one is a response to low-temperature acclimation where the body fluids are in a cooled or supercooled state and the infective juveniles produce trehalose before freezing. During this process glycerol is partly converted to trehalose. The second strategy is a rapid response to freezing which induces the production of glycerol but trehalose levels do not change. Cryoprotective dehydration produces high concentrations of both trehalose and glycerol, suggesting that both an acclimation and a freezing response are triggered. Both glycerol and trehalose act as cryoprotectants in this species and play an important role in its freezing tolerance.
